# Smoke-free vehicles: impact of legislation on child smoke exposure across three countries

**DOI:** 10.1183/13993003.04600-2020

**Published:** 2021-12-02

**Authors:** Anthony A. Laverty, Filippos T. Filippidis, Jasper V. Been, Frances Campbell, Hazel Cheeseman, Nicholas S. Hopkinson

**Affiliations:** 1 Public Health Policy Evaluation Unit, School of Public Health, Imperial College London, London, UK; 2 Division of Neonatology, Dept of Paediatrics, Erasmus MC-Sophia Children's Hospital, University Medical Centre Rotterdam, Rotterdam, The Netherlands; 3 Dept of Public Health, Erasmus MC, University Medical Centre Rotterdam, Rotterdam, The Netherlands; 4 Action on Smoking and Health, London, UK; 5 National Heart and Lung Institute, Imperial College London, London, UK

## Abstract

Second-hand tobacco smoke is a significant threat to the health of children [1]. Across Europe, 12% of children are regularly exposed, a percentage that has stalled in the last decade [2]. In addition to placing children at greater risk of health complications, such as asthma attacks and respiratory tract infections [1], exposure to smoking behaviour by family and peers increases the likelihood of tobacco smoking uptake [3].

## Abstract 2

**Legislation to require no tobacco smoking in vehicles with children is effective in reducing exposure to second-hand smoke** https://bit.ly/3z8fZRa

*To the Editor*:

Second-hand tobacco smoke is a significant threat to the health of children [1]. Across Europe, 12% of children are regularly exposed, a percentage that has stalled in the last decade [2]. In addition to placing children at greater risk of health complications, such as asthma attacks and respiratory tract infections [1], exposure to smoking behaviour by family and peers increases the likelihood of tobacco smoking uptake [3].

Bans on smoking in indoor public places have proven effective in reducing tobacco smoke exposure and improving health in both adults and children [4, 5]. World Health Organization (WHO) guidance recommends extending such legislation to other locations and some European countries have recently banned smoking inside cars when children are present, including England and Wales, Scotland, France, Greece, Ireland and Italy. The evidence base on the policy is limited, with a recent systematic review identifying only five studies of the issue, disagreement about the impacts of the policy and no evaluations of implementation in Scotland [6]. To investigate this further, we use data from a nationally representative survey, conducted across three countries (England, Wales and Scotland) implementing smokefree vehicle policies at different times, to clarify their effect.

Smoking in private vehicles carrying anyone under 18 years became illegal in England and Wales from 1 October, 2015 and in Scotland on 5 December, 2016. Data on exposure came from the Smokefree GB-Youth Survey national internet surveys conducted annually in England, Wales and Scotland by YouGov (a public limited company, London, UK) on behalf of Action on Smoking and Health (ASH) from 2013 to 2020. For these, YouGov recruited participants aged 11–18 years using proprietary software to recruit representative samples described in detail elsewhere [7]. If children were sampled in more than 1 year, we included responses only from when they were first included in the survey. For participants aged <16 years, informed consent was provided by parents, and by the respondents themselves if they were aged 16 to 18 years. We excluded 18 year olds from our analyses.

Our primary outcome was reporting being ever exposed to tobacco smoke in a car. This was based on the question “How often, if at all, do you travel in a car in which someone is smoking?” The question did not specify a time frame for response. Potential answers were: every day; most days; some days; on the odd day; never. We categorised all but “never” as ever exposed. YouGov provided survey weights to make estimates representative of age, sex and region of Great Britain.

We analysed data within an interrupted time series framework and used logistic regression to assess changes in odds of exposure to second-hand smoke in cars among children. We did not use ordered regression as the Brant test indicated violation of the parallel odds assumption (p<0.001). We controlled for country (England and Wales *versus* Scotland), age and sex of children, and a marker of social class based on the job of the chief income earner of the household, classified using the National Statistics Socio-Economic Classification. We have separated this into managerial/professional/supervisory roles *versus* manual/routine/casual occupations. Our analyses fit a linear term for trends before policy implementation, a binary indicator for immediate policy impacts, and a further linear term for trends after implementation.

Sample size was 13 986 children, 1327 in Scotland. Mean±sd age was 14.2±2.0 years and 50.7% were female. Reported levels of exposure to smoking in cars have been falling over time. In 2013, 19.6% of children reported ever being exposed in England and Wales and 25.1% in Scotland. By 2020, these figures had fallen to 14.0% in England and Wales, and 12.5% in Scotland.

Exposure was falling before policy implementation (table 1) (adjusted odds ratio (aOR) 0.86, 95% CI 0.79–0.94) and implementation was associated with a 22% decrease in addition to this trend (aOR 0.78, 95% CI 0.63–0.96). Trends were stable after policy implementation (aOR 1.02, 95% CI 0.97–1.07). Stratified analyses only identified statistically significant declines in exposure associated with policy implementation among girls, children aged 11–14 years old and less deprived children.

**TABLE 1:** Results from interrupted time series logistic regression analyses of impact of policy implementation on self-reported exposure to smoking in vehicles

	**aOR**	**95% CI**	**p-value**
**Policy implementation**	0.78	0.63 to 0.96	0.022
**Annual trend pre policy**	0.86	0.79 to 0.94	0.001
**Annual trend post policy**	1.02	0.97 to 1.07	0.493
**Country (Scotland *versus* England/Wales)**	1.08	0.90 to 1.29	0.420
**Age (years)**	1.16	1.13 to 1.19	<0.001
**Female (*versus* male)**	0.95	0.86 to 1.05	0.296
**NSSEC^#^ deprivation group (more *versus* less)**	1.54	1.39 to 1.71	<0.001
**Stratified analyses**			
**Policy implementation among boys only (n=6902)**	1.00	0.73 to 1.36	0.983
**Policy implementation among girls only (n=7084)**	0.60	0.44 to 0.80	0.001
**Policy implementation among 11–14 year olds (n=7644)**	0.57	0.42 to 0.77	<0.001
**Policy implementation among 15–17 year olds (n=6342)**	1.01	0.75 to 1.36	0.940
**Policy implementation among less deprived children (n=9457)**	0.75	0.56 to 0.98	0.038
**Policy implementation among more deprived children (n=4529)**	0.83	0.59 to 1.17	0.289

Results from fully adjusted regression model controlling for country, age (in years), sex and a marker of deprivation. aOR: adjusted odds ratio. ^#^: social grade based on the National Statistics Socio-Economic Classification (NSSEC), which is a measure of the occupation of the chief income earner of the household.

The impact of policy implementation was sustained when analysis was restricted to children who did not report smoking themselves (aOR 0.72, 95% CI 0.54–0.95; n=11 405) and those who did not report being exposed to smoking inside the home (aOR 0.73, 95% CI 0.57–0.95; n=12 279).

These data support the hypothesis that the ban on smoking in cars with children present has had the intended effect of reducing children's exposure to second-hand smoke, and estimates a 22% relative reduction in exposure. We have used data collected from three separate countries which implemented the policy at two different time points, adding to previous evidence on the impact of such legislation.

The present study uses data from three countries within a quasi-experimental analytical design, with consistent assessment of second-hand smoke exposure over time and between countries. It uses data from 2013–2020, meaning that there are three or four time points before the intervention and an assessment of longer-term changes afterwards. This study nonetheless has some limitations. It is based on self-reported exposure rather than objective assessment, although objective assessment of exposure inside cars only has not been possible in other work. Relatively small numbers and low rates of exposure to smoking in cars meant that we have relied here on assessing ever exposure as there was limited statistical power to examine more frequent exposure. We also relied on only one data point each year and cannot assess other possible impacts, such as third-hand smoke

Our data add to previous analyses of the impact of this policy in England, which came to discrepant results. One study which compared England in the first year after the ban had a much larger estimated impact (a 72% relative reduction compared with 22% here), although this was based on a sample of 13–15 year olds as well as using a measure of regular exposure [8]. Another analysis, including children aged 8–15 years, did not identify an impact over and above a decreasing trend in exposure. Of note, the point estimate for effect was a 23% reduction, which is similar to what we observed in the present study [9]. In addition, our estimate of a 22% reduction in exposure is consistent with a 26% reduction seen in Canada following implementation [10], although larger than the 12% annual change from a similar policy in California [11].

Enforcement of the ban has been with a “light touch”, with few prosecutions [12], suggesting that the change being reported may be due to introduction of the legislation articulating a new social norm that children should be protected in these contexts. Bans on smoking in cars containing children are popular with the public, with survey data from the UK indicating support from a vast majority of the public, including smokers [13]. However, our stratified analyses only identified impacts among girls, younger and less deprived children, which suggests that extensions to these polices and enhanced enforcement may be beneficial. Governments could, for example, consider extending the ban to all cars, which would make the law simpler to enforce as well as protecting all car occupants from harmful toxins in tobacco smoke. Policies to restrict smoking inside cars are uncommon and these results should encourage legislators in other countries to consider such legislation as part of the “Protect” element of the WHO approach to reduce the harms caused by smoking [14].

## Shareable PDF

10.1183/13993003.04600-2020.Shareable1 This one-page PDF can be shared freely online.
Shareable PDF ERJ-04600-2020.Shareable
