# Supplementary material for: Smoke-free vehicles: impact of legislation on child smoke exposure across three countries
Source: Eur Respir J. 2021 Dec 2;58(6):2004600. doi: 10.1183/13993003.04600-2020 (PMC8637180; doi:10.1183/13993003.04600-2020)
Supplement: Supplementary file 1 [file ERJ-04600-2020.Shareable.pdf]

SHAREABLE PDF

Smoke-free vehicles: impact of legislation on child smoke exposure across three countries

Anthony A. Laverty [1], Filippos T. Filippidis [1], Jasper V. Been [2,3], Frances Campbell [4], Hazel Cheeseman [4] and Nicholas S. Hopkinson [5]

[1]Public Health Policy Evaluation Unit, School of Public Health, Imperial College London, London, UK. [2]Division of Neonatology, Dept of Paediatrics, Erasmus MC-Sophia Children's Hospital, University Medical Centre Rotterdam, Rotterdam, The Netherlands. [3]Dept of Public Health, Erasmus MC, University Medical Centre Rotterdam, Rotterdam, The Netherlands. [4]Action on Smoking and Health, London, UK. [5]National Heart and Lung Institute, Imperial College London, London, UK.

Corresponding author: Anthony A. Laverty (a.laverty@ic.ac.uk)

Check for updates

Shareable abstract (@ERSpublications)
Legislation to require no tobacco smoking in vehicles with children is effective in reducing exposure to second-hand smoke https://bit.ly/3z8fZRa

Cite this article as: Laverty AA, Filippidis FT, Been JV, *et al*. Smoke-free vehicles: impact of legislation on child smoke exposure across three countries. *Eur Respir J* 2021; 58: 2004600 [DOI: 10.1183/13993003.04600-2020].

This single-page version can be shared freely online.

Copyright ©The authors 2021.

This version is distributed under the terms of the Creative Commons Attribution Licence 4.0.

Received: 22 Dec 2020
Accepted: 7 Sept 2021

To the Editor:

Second-hand tobacco smoke is a significant threat to the health of children [1]. Across Europe, 12% of children are regularly exposed, a percentage that has stalled in the last decade [2]. In addition to placing children at greater risk of health complications, such as asthma attacks and respiratory tract infections [1], exposure to smoking behaviour by family and peers increases the likelihood of tobacco smoking uptake [3].
